# Exploring Heart Rate Variability Biofeedback as a Nonpharmacological Intervention for Enhancing Perioperative Care: A Narrative Review

**DOI:** 10.4274/TJAR.2024.241658

**Published:** 2024-09-17

**Authors:** Nirupa Ramakumar, Sonu Sama

**Affiliations:** 1 Himalayan Institute of Medical Sciences, Department of Anaesthesiology, Uttarakhand, India; 2 Himalayan Institute of Medical Sciences, Department of Critical Care, Uttarakhand, India

**Keywords:** Analgesia, autonomic nervous system, biofeedback, breathing, heart rate, perioperative care

## Abstract

Heart rate variability biofeedback (HRVBF) is a non-invasive therapeutic technique that aims to regulate variability in heart rate. This intervention has promise in mitigating perioperative stress, a critical factor for surgical patient outcomes. This comprehensive review aimed to explore the current evidence on the perioperative role of HRV biofeedback in improving patient outcomes, reducing perioperative stress, enhancing recovery, and optimizing anaesthesia management. A review of the PubMed and Google Scholar databases was conducted to identify articles focused on HRVBF in relation to the perioperative period. Studies were selected using appropriate keywords in English (MeSH). Ample potential applications of HRVBF in clinical anaesthesia have been identified and proven feasible. It is a non-invasive and an easy method an anaesthesiologists has at its disposal with potential utility in reducing perioperative stress, as a tool of optimization of  comorbidities, analgesia supplementation and in predicting catastrophic complications. Although HRVBF has the potential to enhance anaesthesia management and improve patient outcomes, several limitations and challenges must be addressed to maximize its clinical utility. Overcoming these obstacles through research and technological advancements will be crucial for realizing the full benefits of HRVBF in perioperative care.

**Main Points**

• This in-depth review examined the latest research on how heart rate variability biofeedback (HRVBF) can help improve patient outcomes, lower perioperative stress, speed recovery, and make the most of anaesthesia management.

• This review discusses the mechanisms, methods, and benefits of HRVBF in the perioperative setting, as well as the challenges and future directions for implementing this technique in clinical practice.

• By targeting autonomic regulation, stress reduction, and resilience enhancement, HRVBF techniques offer a personalized approach to perioperative care that may lead to improved patient comfort, optimized surgical outcomes, and enhanced recovery.

## Introduction

Heart rate variability biofeedback (HRVBF) is a non-invasive technique that involves training individuals to regulate their heart rate variability through breathing exercises and relaxation techniques. It has emerged as a promising therapeutic intervention in various clinical settings, including the perioperative period. The significance of perioperative stress on the outcomes of surgical patients is often overlooked.^1^ Currently, the main perioperative interventions used to reduce these stress responses are drugs (mainly anaesthetics and painkillers), which can cause a number of problems. Surgery, in addition to anaesthesia and blood transfusion, can cause immunosuppression, leading to infections and other complications.

The central autonomic network, consisting of complex brain connections, regulates the autonomic nervous system (ANS). HRV is a physiological index that portrays the delicate balance of the ANS. High HRV occurs when the parasympathetic nervous system (PNS) is dominant over the sympathetic nervous system (SNS), indicating good autonomic control. Chronic illnesses such as cardiovascular disease, which often reduce HRV, pose challenges for both patients under anaesthesia and anaesthesiologists.^2^ Using a theoretical perspective, several models have been proposed to elucidate the heart-brain connection, revealing its significant effect on psychological responses and overall well-being.^3^ Notably, the neurovisceral model outlines the relationship between HRV and emotion, cognition, and mental health by showing how the prefrontal brain and cardiac vagal tone are linked.^4^

Goessl et al.,^5^ in their meta-analysis, found a decrease in self-reported stress and improvement in cognitive function, supporting the practical use of HRVBF. While the exact mechanism of HRVBF’s effect is still being studied, it has been suggested that enhancing the baroreflex may indicate improved autonomic balance.^6^ As researchers looked for strategies to enhance HRV, they found that biofeedback using paced breathing exercises at slow respiratory rates was a potentially effective strategy to help individuals raise vagally mediated HRV values by triggering a parasympathetic response. In general, breathing at a resonance frequency of 4.5-6.5 cycles per minute (HRV) stimulates the resonant properties of the cardiovascular system, resulting in larger heart rate oscillations and, in individuals, higher HRV and other advantageous effects, including improved gas exchange.^7^

Postsurgical patients with postoperative pain can experience multiple ramifications for their health and quality of life. Despite extensive research in this field, predictors of postoperative pain are lacking. HRV has been shown to predict postoperative outcomes and postoperative pain.^8, 9^ Niu et al.^10^ found that patients with a heart rate >70 and low HRV during anaesthesia had a higher risk of postoperative intensive care unit stay.^10^ There is a need to include general stress optimization strategies in focusing preoperative stressors beyond the canonical preoperative care workup focused on surgery. Non-pharmacological interventions, in addition to usual perioperative medications, can attenuate somatic and emotional stress. Anaesthesiologists can use HRVBF at the point of care to help prevent overdose because it is an inexpensive, secure, and effective intervention. This comprehensive review aimed to explore the current evidence on the perioperative role of HRVBF in improving patient outcomes, reducing perioperative stress, enhancing recovery, and optimizing anaesthesia management.

## Methods

A review of the PubMed and Google Scholar databases was conducted while searching for articles focused on HRVBF in relation to the perioperative period in the last 10 years. Studies were selected using the keywords in English (MeSH) related to “HRVBF”, “HRV anaesthesiology”, “HRVBF regional anaesthesia”, “HRVBF post-operative pain”, “HRV cancer pain”, and “HRVBF stress”. While describing the utility of HRVBF during the perioperative period, articles on adult and paediatric populations were mainly used.

## Results

### HRV is a Physiological Index that Reflects the ANS Balance

HRV is a physiological index of the delicate balance of the ANS. High HRV was observed when the PNS was predominant over the SNS, indicating good autonomic control. Analysis of HRV revealed irregularities in the activity of ANS and was associated with a higher risk of mortality in individuals with systemic diseases.^11, 12, 13^ The impact of chronic illnesses, particularly cardiovascular disease, which is often associated with HRV, is widely recognized as a major obstacle for both the anaesthetist and the anaesthesiologist.^2^

The vagal nerve, which is classified as the tenth cranial nerve, is a major regulator of the body system. Indeed, there is a correlation between vagal nerve activity and other possible processes and variables that lead to pain, including age and anxiety. The holistic theory of vagal nerve pain modulation established by Gitler et al.^14^ and De Couck et al.^15^ emphasizes the protective role of the vagus nerve in several pain-related processes. These mechanisms include inflammation, abnormal SNS activity, and cellular oxidative stress.

In addition, when the ventral periaqueductal gray, a part of the pain brain circuitry, is stimulated at the brainstem level, it leads to a decrease in pain.^16^ Further research should investigate whether alterations in brain activity patterns account for the reported association between HRV and pain.

Studies using imaging techniques and resting-state functional connectivity (RSFC) have supported the significance of the relationship between the medial prefrontal cortex and limbic regions in heart rate control.^17, 18^ Schumann et al.^18^ conducted a comparative analysis of RSFC patterns among distinct groups of healthy individuals with varying levels of heart rate regulation in a recent paper. The findings of this study suggest that individuals with slower heart rates have a notable increase in functional connectivity (RSFC) within a functional network encompassing multiple regions of the central nervous system (CNS) compared with individuals with faster heart rates.

HRV is derived from electrocardiogram (ECG) readings, and HRV variables are collected in both the temporal and spectral domains. Time-domain variables, such as the root mean square of successive deviations between normal heartbeats (rMSSD), are usually the best and fastest way to measure changes in HRV caused by changes in the vagus nerve.^19^ rMSSD is the primary characteristic utilized in mobile HRV applications owing to its ease of acquisition and computation using brief time periods.^20^

### Mechanisms Through Which HRV Biofeedback Training Affects the ANS

**Figure 1:**
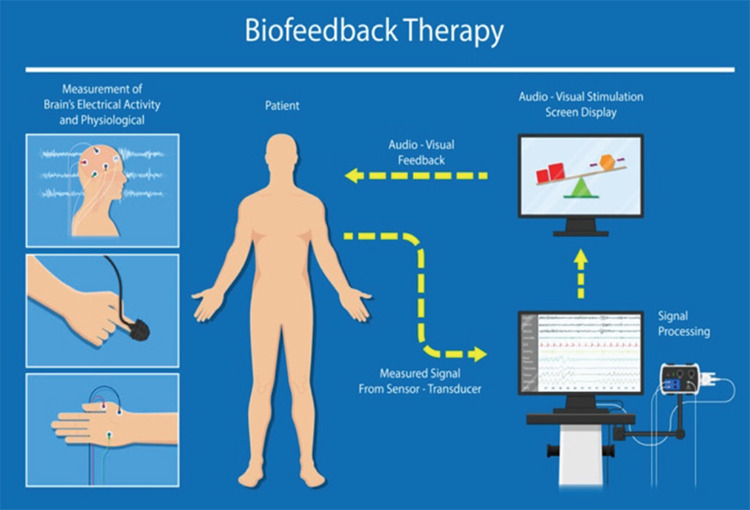
BIOFEEDBACK. Positive Outcomes, Inc. Accessed on May 30, 2024. https://positiveoutcomesllc.com/test-page/

The HRV BF technique incorporates breathing elements and delivers data in the form of a customized digital interface.^21^ The individual was positioned on a pulse monitor or ECG lead, and the resulting pulse or ECG tracing, as well as the intervals between beats, were shown on a computer monitor (Figure 1).

Decreased respiration amplifies HRV, and when the breathing rate reaches approximately six breaths per minute, the pattern becomes more pronounced and takes the shape of a sine wave. This phenomenon is commonly referred to as “resonance” or “coherence”, and it can be quantified using mathematical methods and visually perceived.^22^ The patient is advised to cultivate this pattern through deliberate breathing while also invoking a sense of calm.

**Figure 2:**
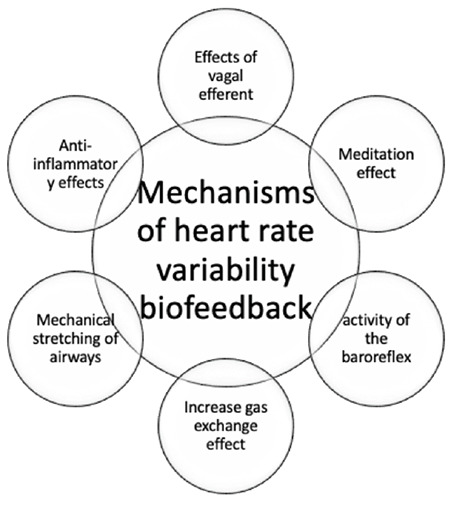
Schematic representation of the various mechanism of HRVBF. HRVBF, Heart rate variability biofeedback.

It is widely accepted that the resonant pattern is mostly caused by increased heart rate stimulation through vagal mechanisms, namely the “brake and release” response, which occurs in coordination with respiration. It is widely acknowledged that HRV BF promotes breathing by enhancing vagal tone and promoting a calm state. Figure 2 schematizes the various mechanisms of HRVBF.

### Specific Physiological and Neural Pathways Involved in HRVBF

The influence of biofeedback on brain function is unclear. Participants stimulated the primary vagal reflexes, specifically the baroreflex by modifying their breathing patterns to enhance heart rate oscillations.^23^ The baroreflex is an extremely effective mechanism for regulating heart rate in the immediate term.  

HRVBF might improve the input from the vagus nerve, which would then stimulate the cardiovagal brainstem nuclei in a manner similar to direct electrical stimulation. Vagal nerve stimulation affects both the central autonomic network and limbic system by modulating vagal afferent activity.^24^ The nucleus of the solitary tract acts as a central hub for integrating sensory information from the periphery.^25^ As part of this, signals are sent to the noradrenergic and serotonergic neuromodulator systems, and baroreceptors and lung stretch receptors process the information received.

A recent meta-analysis demonstrated the anxiety-reducing efficacy of both continuous HRVBF and one-time HRVBF.^5, 26^ One session of HRVBF has been shown to be beneficial for individuals with posttraumatic depression. Physiological coherence refers to the extent to which rhythmic activity within living systems exhibits peace, equilibrium, and steadiness within a specific timeframe.^6^ The objective of HRVBF is to attain elevated physiological coherence with a higher level of proficiency resulting in such coherence. Scientists have observed that activating vagal afferent pathways during high physiological coherence can change parts of the brain that control emotions. These include the locus coeruleus, orbitofrontal cortex, insula, hippocampus and amygdala.^7^

### HRVBF Methods

In their systematic review, Lalanza et al.^22^ described different protocols and method-related limitations. They also proposed a checklist to improve protocol quality. The three HRVBF protocols depend on the presence or absence of a previously detected resonant frequency (RF). Breathing in the range of 6.5-4.5 b m for 2 min at a time can detect RF. The participants or patients receive a special device that monitors and displays their heart rate. 

**1. Biofeedback devices:** Specialized biofeedback devices are used to measure variability in heart rate and provide real-time feedback to patients. These devices typically consist of sensors that monitor the heart rate and software that processes the data and presents them in a user-friendly manner. Patients can observe changes in their heart rate variability patterns and learn to modulate their autonomic responses through guided exercises. Few studies have demonstrated that wearable devices display good correlation with ECG-based HRV measurements in terms of comfort, robustness, and non-invasiveness.^27^

**2. Breathing techniques:** Controlled breathing exercises are fundamental to HRVBF. Patients will be guided to practice slow, deep breathing patterns that can help regulate variability in heart rate and induce a state of relaxation. By synchronizing breathing with specific HRV parameters, patients can enhance their vagal tone and achieve a balanced autonomic state.^28^ Deep breathing was found to be useful in lowering preoperative anxiety in 40% of presurgical patients.^29^

**3. Visual and auditory feedback:** Biofeedback devices often provide visual or auditory cues to help patients regulate their heart rate variability. For example, patients may be instructed to match their breathing rate with a visual representation of their heart rate variability on a screen or by following auditory cues, such as tones or sounds that change based on their physiological state.^30^ Muzzi et al.^31^ evaluated the effects of intraoperative auditory stimulation on postoperative pain in children undergoing adenotonsillectomy. The researchers found a clinically significant reduction in postoperative pain and emergence delirium in children.

**4. Guided imagery and relaxation techniques:** Incorporating guided imagery and relaxation techniques into HRVBF sessions can enhance the effectiveness of the intervention.^32^ Patients may be guided to visualize calming scenes or engage in progressive muscle relaxation exercises to promote relaxation, reduce anxiety, and improve their overall emotional well-being during the perioperative period.^33, 34^ It can be used as a complementary treatment to postoperative pain management in all patients.

### Benefits of HRVBF in the Perioperative Setting

**1. Stress reduction: **Research suggests that HRVBF can effectively reduce perioperative stress and anxiety levels in surgical patients by promoting relaxation, enhancing parasympathetic activity, and mitigating the physiological stress response to surgery and anaesthesia. The authors found significantly higher preoperative anxiety in day care (34%) and inpatients (38.3%) posted for day care surgery.^35^ Amalan et al.^36^ investigated HRV-based stress detection and demonstrated similarity in patients’ pre-surgery stress. Use has been demonstrated in obstetric patients for stress reduction in the postpartum period and in patients undergoing the first in vitro fertilization with embryo transfer.^37^ van der Zwan et al.^38^ examined how well self-hel pphysical activity (PA), mindfulness meditation (MM), and HRVBF helped 76 healthy volunteers deal with stress and its associated symptoms.^38^ They found that HRVBF was as good as PA and MM for reducing stress and associated symptoms. Reduced stress levels not only improve patient comfort but may also positively impact surgical outcomes and recovery. A meta-analysis by Pizzoli et al.^21^ showed that HRVBF is effective for improving mental well-being. HRVBF was used to effectively reduce blood pressure in 43 prehypertensive patients by Lin et al.^39^ We can extrapolate the study findings to hypertensive patients who undergo elective surgery in a pre-anaesthetic clinic to utilize HRVBF as a non-pharmacological intervention to optimize blood pressure and hence improve patient outcomes. HRVBF is associated with decreased stress levels in peripartum women, highlighting its potential as an adjuvant treatment for stress management during the peripartum period.^40^ Moreover, in patients with cardiovascular disease, HRVBF has been linked to lower rates of all-cause readmissions, improved 6-min walk test results, and reduction in blood pressure.^41^ HRVBF can be an effective tool to mitigate perioperative stress levels and improve overall well-being.

**2. Pain management:** HRVBF has shown promise as a complementary approach to perioperative pain management. By changing autonomic function, raising vagal tone, and encouraging relaxation, HRVBF techniques can help lower pain perception, opioid use, and postoperative pain medication needs, leading to better pain control and faster recovery.

These studies have shown that patients with postoperative pain have lower HRV.^42, 43^ The authors did not keep track of the pain score trends, which limited the study limitations due to their sampling methods (cross-sectional design). This systematic review supports an inverse relationship between HRV and pain, as shown in pragmatic studies.^44^ This study also validates a CNS modulatory basis for the effects of vagal nerve stimulation on pain.

Anderson et al.^45^ conducted a prospective observational study of 65 patients scheduled for laparoscopic cholecystectomy. They found that changes in the high-frequency HR variability index indicated changes in the balance between pain and analgesia.^45^ Intraoperative titrate analgesia may help individual patients. Girishan Prabhu et al.^46^ aimed to compare the efficacy of nature-based virtual reality (VR) and HRVBF in reduce surgical postoperative pain and anxiety. They randomly enrolled 30 patients undergoing total knee arthroplasty into three groups: control, video-assisted HRVBF, and VR with HRVBF. They found that both groups had greater PNS activity levels, and VR with HRVBF mitigated pain more than VR with HRVBF alone (*P *< 0.01). It would be beneficial to identify patients with anxiety during the preoperative period using appropriate questionnaires. It becomes the responsibility of the anaesthetist to take care of the stress, as it can have various far-reaching consequences, even in the postoperative period. VR interventions, often combined with other techniques, such as active communication and HRVBF, can effectively reduce pain and anxiety in children and adolescents undergoing various medical procedures, including surgery.^47, 48^

**3. Anaesthetic management:** The potential impact of HRVBF on optimizing anaesthetic management during the perioperative period is worth noting. HRVBF interventions improve hemodynamic stability, anaesthesia depth modulation, and perioperative outcomes by changing vagal tone, physiological coherence, and autonomic balance. This approach has improved patient safety and perioperative care. Patients who experienced the adaptive VR-based HRVBF environment reported significant decreases in preoperative anxiety and postoperative pain after VR intervention.^49^

The depth of anaesthesia is an essential component of standard anaesthesia monitoring to prevent intraoperative awareness. It ensures safe and high-quality anaesthesia, thereby decreasing anaesthesia-related complications. HRV correlates well with anaesthesia depth.^50^ Zhan et al.^50^ developed an ingenious method for distinguishing various states of anaesthesia based on HRV-derived features in combination with a deep neural network.

High-frequency HRV as a marker of nociception-analgesia balance is a better choice than other usual hemodynamic changes.^51^ Analgesia nociception index (ANI) and high-frequency variability index monitors (Mdoloris Medical Systems) were used to consider the HRV value.^52^ The major limitation of this study is that ANI in the awake state is not conclusive because of the profound effect of the patient’s emotional status.

**4. Recovery and rehabilitation:** Preliminary evidence suggests that HRVBF interventions could play a significant role in facilitating postoperative recovery and rehabilitation.^53, 54^ By promoting adaptive stress responses, enhancing resilience, and supporting physiological coherence, HRVBF may facilitate faster recovery, improved functional outcomes, and enhanced overall well-being during the postoperative period. Additionally, HRV-BF has shown promise in decreasing anxiety, improving HRV, and enhancing vasomotor function in patients with alcohol dependence, thereby complementing standard rehabilitative care.^55^ A systematic review by Burlacu et al.^41^ demonstrated the beneficial effects of HRVBF on various cardiovascular diseases. HRVBF can be complementary to improving postoperative outcomes in cardiac patients who undergo cardiac and non-cardiac surgery.

There is a positive relationship between increased HRV and traumatic brain injury recovery following biofeedback, including improvements in cognitive and emotional functioning and physical symptoms, such as headaches, dizziness, and sleep problems.^56, 57^ Anaesthesiologists, in collaboration with surgical and psychological teams, facilitated the rehabilitation of postsurgical patients using HRVBF.^58^ Oncological patients frequently undergo resection for recurring tumors, especially head and neck and breast cancer. It is beneficial to reduce stress and anxiety levels to help anaesthesiologists better manage pain and achieve better functional outcomes. HRVBF can help alleviate chronic pain in cancer survivors.^59, 60, 61^ HRVB training can improve HRV coherence ratios among cancer survivors, thereby improving cancer-related symptom management.

### Challenges, Limitations, and Future Directions for the Implementation of HRVBF Training in Clinical Settings

Although variability in HRVBF holds promise in the management of anaesthesia, there are several limitations and challenges associated with its use in clinical practice. Some key limitations of the proposed model include the following.

**1. Training and expertise:** Implementing HRVBF in anaesthesia management requires specialized training from healthcare providers, including anaesthesiologists and nursing staff. Not all medical professionals have the necessary expertise to effectively interpret HRV data and integrate it into anaesthesia care. This could potentially limit the widespread adoption of HRVBF in the clinical setting.^61^ Low compliance rates in the study group and poor feasibility are some methodological limitations that researchers can face.^62^

**2. Equipment and technology:** HRVBF typically relies on the use of specialized equipment and technology to monitor and analyze HRV. Access to such devices may be limited in certain healthcare settings, particularly in resource-limited environments or in smaller facilities. The cost of acquiring and maintaining this technology could also be a barrier to its widespread implementation. Ectopic beats, other abnormal heart rhythms, lines, movements, or electromyogram artifacts are just a few examples of factors that can make it difficult to accurately detect RR intervals.^63^ When conducting HRV data collection, studies should consider factors such as noise, temperature, illumination, humidity, time, and participant postures. It is crucial to have a strategy in place for preventing artifacts and analyzing data before commencing an HRV study. Complex, non-categorical values can impede the routine clinical application of HRV.^8^ Li et al.^64^ presented a technique that uses white noise to mimic the interference that wearable technology can experience in everyday situations to assess the accuracy of these devices. Further research is required in this field.

**3. Standardization and guidelines:** There are currently no standardized protocols and guidelines for the use of HRVBF in anaesthesia management. The absence of clear recommendations on how to incorporate HRV data into clinical decision-making may hinder its effective and consistent application across different healthcare settings.^65^ Further research is needed to establish best practices and evidence-based guidelines for HRVBF in anaesthesia care.

**4. Patient variability:** Individual differences in patient responses to HRVBF may pose challenges in its application in anaesthesia management. Not all patients benefit equally from this technique, and factors such as age, comorbidities, and baseline physiological state can influence the effectiveness of HRVBF interventions. According to the results of a cohort study involving 167,548 people (aged 6 months to 93 years), HRV sharply decreased with age until approximately 60 years of age, at which point it stabilized.^66^ Conversely, Lehrer et al.^67^ found that age-associated obliteration of biofeedback changes on HRV had no effect on the efficacy of the HRVBF method in their research population involving geriatric asthmatic patients. Tailoring HRV biofeedback to each patient’s unique characteristics and needs is essential to maximize its benefits.

**5. Interpretation and integration:** Interpreting HRV data and integrating them into clinical decision-making processes can be complex and time-consuming. Anaesthesiologists and healthcare providers must have the knowledge and skills to effectively analyze HRV parameters and translate this information into actionable insights for optimizing anaesthesia management. This process may require additional resources and training to ensure accurate and meaningful HRVBF.

**6. Ethical and privacy concerns:** The collection and analysis of physiological data through HRVBF raises ethical considerations related to patient privacy and data security. Safeguards must be in place to protect patient information and ensure compliance with data protection regulations. Healthcare providers must also communicate transparently with patients regarding the purpose and implications of HRVBF.

In summary, although HRV biofeedback has the potential to enhance anaesthesia management and improve patient outcomes, several limitations and challenges must be addressed to maximize its clinical utility. Overcoming these obstacles through research, education, and technological advancements will be crucial for realizing the full benefits of HRV biofeedback in perioperative care.

### Future Directions

Individualized training programs are essential for optimizing the benefits of HRVBF during the perioperative period. Healthcare providers can tailor biofeedback protocols according to each patient’s specific needs, considering factors such as baseline HRV levels, medical history, and surgical procedure. Customized training programs can enhance patient engagement and adherence to HRVBF intervention.

**Figure 3:**
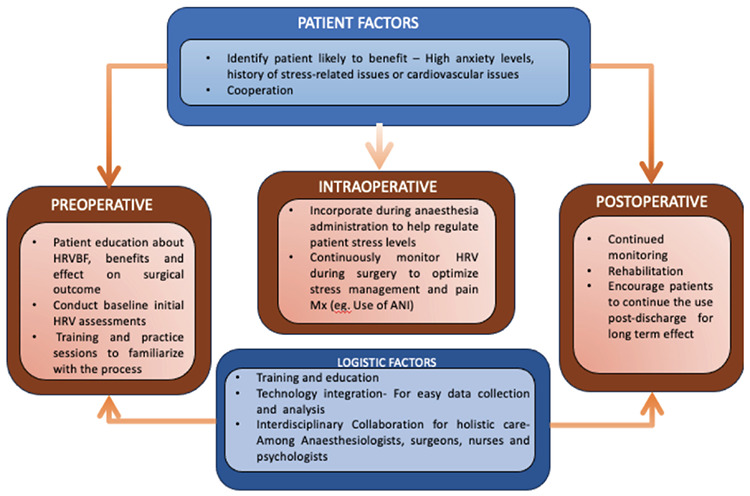
Perioperative care model for the use of HRVBF for patient management. ANI, analgesia nociception index; HRVBF, Heart rate variability biofeedback.

HRVBF can be integrated into anaesthesia management protocols to optimize perioperative patient outcomes. A Perioperative care model is suggested for the use of HRVBF for patient management (Figure 3). Anaesthesiologists can use HRV data to adjust anaesthetic dosages, monitor patient stress levels, and personalize anaesthetic care based on individual autonomic responses. Collaboration between anaesthesia providers and biofeedback specialists is essential for the seamless integration of HRVBF into perioperative care.

Continuation of HRVBF during the postoperative period can promote recovery, reduce pain, and enhance patient well-being. Follow-up sessions and remote monitoring can help patients sustain positive outcomes and effectively manage postoperative stress.

## Conclusion

While the accuracy of this approach may be uncertain in this scenario, if anaesthesiologists possess a tool capable of consistently evaluating HRV in real time may potentially employ it to adjust management. In conclusion, HRVBF is a valuable tool for perioperative care, improving patient outcomes, recovery, and anaesthesia management. By targeting autonomic regulation, stress reduction, and resilience enhancement, HRVBF techniques offer a personalized approach to perioperative care that may improve patient comfort, optimize surgical outcomes, and enhance recovery. To maximize HRVBF and advance personalized care in the perioperative setting, more research and clinical integration are needed. These improvements will ultimately lead to better patient outcomes and higher quality of care in surgical practice.

## References

[1] Gan TJ (2017). Poorly controlled postoperative pain: prevalence, consequences, and prevention.. J Pain Res.

[2] Zawadka M, Marchel M, Andruszkiewicz P (2020). Diastolic dysfunction of the left ventricle - a practical approach for an anaesthetist.. Anaesthesiol Intensive Ther.

[3] Aristizabal JP, Navegantes R, Melo E, Pereira A Jr (2020). Use of heart rate variability biofeedback to reduce the psychological burden of frontline healthcare professionals against COVID-19.. Front Psychol.

[4] Thayer JF, Lane RD (2000). A model of neurovisceral integration in emotion regulation and dysregulation.. J Affect Disord.

[5] Goessl VC, Curtiss JE, Hofmann SG (2017). The effect of heart rate variability biofeedback training on stress and anxiety: a meta-analysis.. Psychol Med.

[6] McCraty R, Zayas MA (2014). Cardiac coherence, self-regulation, autonomic stability, and psychosocial well-being.. Front Psychol.

[7] Lehrer P, Kaur K, Sharma A (2020). Heart rate variability biofeedback improves emotional and physical health and performance: a systematic review and meta analysis.. Appl Psychophysiol Biofeedback.

[8] Lakshmi SKSD, Nanda A, Pakhare V. Impact of heart rate variability on anesthesia, critical care and pain.New Advances in Medicine and Medical Science. :3;150-165..

[9] Frandsen MN, Mehlsen J, Foss NB, Kehlet H Preoperative heart rate variability as a predictor of perioperative outcomes: a systematic review without meta-analysis.. Journal of clinical monitoring and computing.

[10] Niu J, Lu Y, Xu R (2023). The prognostic value of intraoperative HRV during anesthesia in patients presenting for non-cardiac surgery.. BMC Anesthesiol.

[11] Drawz PE, Babineau DC, Brecklin C (2013). Heart rate variability is a predictor of mortality in chronic kidney disease: a report from the CRIC Study.. Am J Nephrol.

[12] Wujtewicz M, Owczuk R (2023). Heart rate variability in anaesthesiology - narrative review.. Anaesthesiol Intensive Ther.

[13] França da Silva AK, Penachini da Costa de Rezende Barbosa M, Marques Vanderlei F, Destro Christofaro DG, Marques Vanderlei LC (2016). Application of heart rate variability in diagnosis and prognosis of individuals with diabetes mellitus: systematic review.. Ann Noninvasive Electrocardiol.

[14] Gitler A, Vanacker L, De Couck M, De Leeuw I, Gidron Y (2022). Neuromodulation applied to diseases: the case of hrv biofeedback.. J Clin Med.

[15] De Couck M, Caers R, Musch L, Fliegauf J, Giangreco A, Gidron Y (2019). How breathing can help you make better decisions: Two studies on the effects of breathing patterns on heart rate variability and decision-making in business cases.. Int J Psychophysiol.

[16] Pereira EAC, Lu G, Wang S, et al. Ventral periaqueductal grey stimulation alters heart rate variability in humans with chronic pain.Exp Neurol.2010 Jun;223(2):574-581..

[17] Kumral D, Schaare HL, Beyer F (2019). The age-dependent relationship between resting heart rate variability and functional brain connectivity.. Neuroimage.

[18] Schumann A, de la Cruz F, Köhler S, Brotte L, Bär KJ (2021). The influence of heart rate variability biofeedback on cardiac regulation and functional brain connectivity.. Front Neurosci.

[19] Shaffer F, Ginsberg JP (2017). An Overview of Heart Rate Variability Metrics and Norms.. Front Public Health.

[20] Penttilä J, Helminen A, Jartti T (2001). Time domain, geometrical and frequency domain analysis of cardiac vagal outflow: effects of various respiratory patterns.. Clin Physiol.

[21] Pizzoli SFM, Marzorati C, Gatti D, Monzani D, Mazzocco K, Pravettoni G (2021). A meta-analysis on heart rate variability biofeedback and depressive symptoms.. Sci Rep.

[22] Lalanza JF, Lorente S, Bullich R, García C, Losilla JM, Capdevila L (2023). Methods for heart rate variability biofeedback (HRVB): a systematic review and guidelines.. Appl Psychophysiol Biofeedback.

[23] Lehrer PM, Gevirtz R (2014). Heart rate variability biofeedback: how and why does it work?. Front Psychol.

[24] Fang Y-T, Lin Y-T, Tseng W-L (2023). Neuroimmunomodulation of vagus nerve stimulation and the therapeutic implications.. Front Aging Neurosci.

[25] Zoccal DB, Furuya WI, Bassi M, Colombari DS, Colombari E (2014). The nucleus of the solitary tract and the coordination of respiratory and sympathetic activities.. Front Physiol.

[26] Saito R, Sawamura D, Yoshida K, Sakai S (2021). Relationship between the proficiency level and anxiety-reducing effect in a one-time heart rate variability biofeedback: A randomized controlled trial.. Medicine (Baltimore).

[27] Sammito S, Böckelmann I (2016). Möglichkeiten und Einschränkungen der Herzfrequenzmessung und der Analyse der Herzfrequenzvariabilität mittels mobiler Messgeräte: Eine systematische Literaturübersicht [Options and limitations of heart rate measurement and analysis of heart rate variability by mobile devices: A systematic review].. Herzschrittmacherther Elektrophysiol.

[28] Lombardi F, Stein PK (2011). Origin of heart rate variability and turbulence: an appraisal of autonomic modulation of cardiovascular function.. Front Physiol.

[29] Toussaint L, Nguyen QA, Roettger C (2021). Effectiveness of progressive muscle relaxation, deep breathing, and guided imagery in promoting psychological and physiological states of relaxation.. Evid Based Complement Alternat Med.

[30] Demirci H, van der Storm SL, Huizing NJ (2023). Watching a movie or listening to music is effective in managing perioperative anxiety and pain: a randomised controlled trial.. Knee Surg Sports Traumatol Arthrosc.

[31] Muzzi E, Ronfani L, Bossini B, Lezcano C, Orzan E, Barbi E (2021). Effects of intraoperative auditory stimulation on pain and agitation on awakening after pediatric adenotonsillectomy: a randomized clinical trial.. JAMA Otolaryngol Head Neck Surg.

[32] Felix MMDS, Ferreira MBG, Oliveira LF, Barichello E, Pires PDS, Barbosa MH (2018). Guided imagery relaxation therapy on preoperative anxiety: a randomized clinical trial.. Rev Lat Am Enfermagem.

[33] Charette S, Fiola JL, Charest M-C (2015). Guided imagery for adolescent post-spinal fusion pain management: a pilot study.. Pain Manag Nurs.

[34] Felix MMDS, Ferreira MBG, da Cruz LF, Barbosa MH (2019). Relaxation therapy with guided imagery for postoperative pain management: an integrative review.. Pain Manag Nurs.

[35] Wetsch WA, Pircher I, Lederer W (2009). Preoperative stress and anxiety in day-care patients and inpatients undergoing fast-track surgery.. Br J Anaesth.

[36] Amalan S, Vaishali B, S P P, Joseph J, Sivaprakasam M (2019). Pre-surgery stress monitoring using heart rate variability measures.. Annu Int Conf IEEE Eng Med Biol Soc.

[37] Bian Y, Liu F, Wang Y, Wang X, Chen R. Effects of heart rate variability (HRV) biofeedback for women undergoing first-time in vitro fertilization and embryo transfer.Altern Ther Health Med. ;29(2):162-167..

[38] van der Zwan JE, de Vente W, Huizink AC, Bögels SM, de Bruin EI (2015). Physical activity, mindfulness meditation, or heart rate variability biofeedback for stress reduction: a randomized controlled trial.. Appl Psychophysiol Biofeedback.

[39] Lin G, Xiang Q, Fu X (2012). Heart rate variability biofeedback decreases blood pressure in prehypertensive subjects by improving autonomic function and baroreflex.. J Altern Complement Med.

[40] Kudo N, Shinohara H, Kodama H Heart rate variability biofeedback intervention for reduction of psychological stress during the early postpartum period.. Applied psychophysiology and biofeedback.

[41] Burlacu A, Brinza C, Popa IV, Covic A, Floria M (2021). Influencing cardiovascular outcomes through heart rate variability modulation: a systematic review.. Diagnostics (Basel).

[42] Chang LH, Ma TC, Tsay SL, Jong GP (2012). Relationships between pain intensity and heart rate variability in patients after abdominal surgery: a pilot study.. Chin Med J (Engl).

[43] Caton L, Bolzon M, Boschiero D, Thayer JF, Gidron Y (2021). Pre-surgical heart-rate variability strongly predicts less post-operative pain in patients with epilepsy.. J Psychosom Res.

[44] Tracy LM, Ioannou L, Baker KS, Gibson SJ, Georgiou-Karistianis N, Giummarra MJ (2016). Meta-analytic evidence for decreased heart rate variability in chronic pain implicating parasympathetic nervous system dysregulation.. Pain.

[45] Anderson TA, Segaran JR, Toda C, Sabouri AS, De Jonckheere J (2020). High-frequency heart rate variability index: a prospective, observational trial assessing utility as a marker for the balance between analgesia and nociception under general anesthesia.. Anesth Analg.

[46] Girishan Prabhu V, Stanley L, Morgan R, Shirley B (2024). Designing and developing a nature-based virtual reality with heart rate variability biofeedback for surgical anxiety and pain management: evidence from total knee arthroplasty patients.. Aging Ment Health.

[47] Fahrenkamp A, Benore E (2019). The role of heart rate variability biofeedback in pediatric chronic pain rehabilitation: A case series design.. Clinical Practice in Pediatric Psychology.

[48] Orgil Z, Karthic A, Bell N (2023). Dataset used to refine a treatment protocol of a biofeedback-based virtual reality intervention for pain and anxiety in children and adolescents undergoing surgery.. Data Brief.

[49] Kothgassner OD, Goreis A, Bauda I, Ziegenaus A, Glenk LM, Felnhofer A (2022). Virtual reality biofeedback interventions for treating anxiety : A systematic review, meta-analysis and future perspective.. Wien Klin Wochenschr.

[50] Zhan J, Wu Z, Duan ZX (2021). Heart rate variability-derived features based on deep neural network for distinguishing different anaesthesia states.. BMC Anesthesiol.

[51] Anderson TA (2017). Heart rate variability: implications for perioperative anesthesia care.. Curr Opin Anaesthesiol.

[52] Yoshida K, Obara S, Inoue S (2023). Analgesia nociception index and high frequency variability index: promising indicators of relative parasympathetic tone.. J Anesth.

[53] Deschamps A, Denault A (2008). Analysis of heart rate variability: a useful tool to evaluate autonomic tone in the anesthetized patient?. Can J Anaesth.

[54] Mazzeo AT, La Monaca E, Di Leo R, Vita G, Santamaria LB (2011). Heart rate variability: a diagnostic and prognostic tool in anesthesia and intensive care.. Acta Anaesthesiol Scand.

[55] Penzlin AI, Siepmann T, Illigens BM, Weidner K, Siepmann M (2015). Heart rate variability biofeedback in patients with alcohol dependence: a randomized controlled study.. Neuropsychiatr Dis Treat.

[56] Talbert LD, Kaelberer Z, Gleave E, et al. A systematic review of heart rate variability (HRV) biofeedback treatment following traumatic brain injury (TBI).Brain Inj. ;37(7):635-642..

[57] Chen H, Wang XT, Ding X (2016). [The correlation between optic nerve sheath diameter and volume status in patients after cardiac surgery].. Zhonghua Nei Ke Za Zhi.

[58] Barth CA, Wladis A, Roy N, Blake C, Kolo SM, O'Sullivan C (2022). Ways to improve surgical outcomes in low- and middle-income countries.. Bull World Health Organ.

[59] Lazaridou A, Edwards RR (2019). Relaxation Techniques and biofeedback for cancer pain management.. Essentials of Interventional Cancer Pain Management.

[60] Burch JB, Ginsberg JP, McLain AC (2020). Symptom management among cancer survivors: randomized pilot intervention trial of heart rate variability biofeedback.. Appl Psychophysiol Biofeedback.

[61] Crevenna R (2022). Biofeedback in medicine with a focus on cancer rehabilitation.. Wien Klin Wochenschr.

[62] Hirten RP, Danieletto M, Landell K (2024). Remote short sessions of heart rate variability biofeedback monitored with wearable technology: open-label prospective feasibility study.. JMIR Ment Health.

[63] Shafqat K, Pal SK, Kumari S, Kyriacou PA. Changes in heart rate variability in patients under local anesthesia.Annual International Conference of the IEEE Engineering in Medicine and Biology Society. IEEE Engineering in Medicine and Biology Society. Annual International Conference.2007;299-302..

[64] Li X, Song Y, Wang H (2023). Evaluation of measurement accuracy of wearable devices for heart rate variability.. iScience.

[65] Kim HJ, Park Y, Lee J (2024). The Validity of Heart Rate Variability (HRV) in Educational Research and a Synthesis of Recommendations.. Educational Psychology Review.

[66] Tegegne BS, Man T, van Roon AM, Riese H, Snieder H (2018). Determinants of heart rate variability in the general population: The Lifelines Cohort Study.. Heart Rhythm.

[67] Lehrer P, Vaschillo E, Lu SE (2006). Heart rate variability biofeedback: effects of age on heart rate variability, baroreflex gain, and asthma.. Chest.

